# Enhancing Shape Sensing of Slender Medical Continuum Robot Using Carbon Nanotube Piezoresistive Fiber Bandage

**DOI:** 10.34133/cbsystems.0622

**Published:** 2026-06-24

**Authors:** Pingyu Xiang, Xiangyu Mi, Hongye Zhang, Fei Wang, Xiong Yang, Yue Wang, Rong Xiong, Song Liu, Haojian Lu

**Affiliations:** ^1^ Department of Control Science and Engineering, Zhejiang University, Hangzhou 310027, China.; ^2^ Department of Electronic and Computer Engineering, The Hong Kong University of Science and Technology, Hong Kong, China.; ^3^ School of Information Science and Technology, ShanghaiTech University, Shanghai 201210, China.; ^4^ Stomatology Hospital, School of Stomatology, Zhejiang University School of Medicine, Zhejiang Provincial Clinical Research Center for Oral Diseases, Zhejiang Key Laboratory of Oral Biomedical, Hangzhou 310000, China.; ^5^ Engineering Research Center of Oral Biomaterials and Devices of Zhejiang Province, Hangzhou 310000, China.

## Abstract

Slender medical continuum robots with flexibility and highly redundant degrees of freedom are widely used in various minimally invasive surgery. However, when interacting with anatomical structures, the continuum robot adopts diverse shapes, posing challenges for operation and control. To achieve real-time intraoperative shape sensing and provide online guidance for manipulation, most existing methods rely on optical fibers embedded within the robot, which often require specialized robot designs and come with high costs. Here, we present a novel approach utilizing thin and flexible carbon nanotube piezoresistive fibers as a bandage, helically integrated on the surface of existing slender medical continuum robots for shape sensing. The spatial configuration of the robot is effectively inferred by downsampling the resistance changes along the robot’s body and applying a learning-based method. The results demonstrate that the proposed helically arranged carbon nanotube piezoresistive fibers, combined with a data-driven approach, are capable of reconstructing the robot’s spatial shape. In vitro and ex vivo experiments on animal tissues further highlight its promising potential for enhancing the shape-sensing ability of existing medical continuum robots.

## Introduction

Slender medical continuum robots (SMCRs), such as steerable needles [1], catheters [2], and flexible endoscopes [3], have gained considerable attention in minimally invasive surgery (MIS) [4]. With their highly redundant degrees of freedom, these robots can actively or passively bend during procedures to conform to complex anatomical structures [5], notably improving patient outcomes compared to open surgeries [6]. However, their remarkable flexibility also presents substantial challenges. When the field of view is obscured by human tissue, accurately determining the robot’s complex shape becomes challenging. Real-time shape sensing of continuum robots is critical for ensuring operational efficiency, providing surgeons with real-time guidance, and facilitating the successful execution of surgical tasks [7]. Numerous studies have explored methods for shape sensing in soft continuum robots [8,9]. Most existing approaches rely on markers attached to the robot’s body, reconstructing the shape by position [10], curvature [11], or strain [12] at these marker points. Other methods involve direct visualization of the robot’s shape through cameras [13] or intraoperative fluoroscopy [14]. While offering high accuracy and more intuitive results, they face substantial limitations of being restricted by the surgical field’s line-of-sight constraints or the harmful ionizing radiation introduced by fluoroscopy. Thus, we advocate for an indirect reconstruction approach to continuum robot shape sensing, which avoids exposure to ionizing radiation, making it a safer and more feasible solution for clinical applications.

Electromagnetic tracking sensors, which have been widely adopted and commercialized, serve as a powerful tool for shape sensing in numerous studies on continuum robots. Miniature coils are discretely placed along the robot’s body, decoding the magnetic fields generated by an external field generator to determine the position and orientation in space. Combining the discrete pose information from the coils with the kinematic model of the robot, the spatial shape of the robot can be effectively reconstructed [10,15,16]. Ultrasound imaging offers another radiation-free approach for shape reconstruction [17,18]. While ultrasound scanning provides depth information, its imaging quality is affected by the surrounding tissue, leading to a substantial reduction in reconstruction accuracy. In some cases, ultrasound images need to be fused with x-ray fluoroscopy to achieve better results, where associated registration and denoising algorithms often compromise real-time performance. In addition to reconstructing the shape of the continuum robot itself, there is also the need to estimate the shape of surgical instruments, such as biopsy forceps and probes that extend from the working channel of the robot. In these scenarios, direct optical imaging via endoscopes provides an ideal solution [19,20].

Compared to shape reconstruction methods such as electromagnetic sensors, ultrasound imaging, and intraoperative x-ray fluoroscopy, which require additional devices as external references, shape reconstruction based on fiber Bragg gratings (FBGs) offers a more straightforward deployment [7,11,21–23]. By detecting wavelength shifts in reflected light from the gratings, FBGs directly measure the strain and curvature at corresponding segments of the robot’s body. This approach enables highly accurate shape estimation, and the slender optical fibers embedded in the robot have minimal impact on its stiffness characteristics. However, FBG-based sensing typically necessitates a specialized design of the robot from the outset, including preallocated channels for fiber installation. In addition, FBG sensors are relatively expensive, and their low ductility makes them prone to breakage under large bending angles, which limits their robustness in certain applications.

Resistive strain sensors are known for their high sensitivity and low cost. However, conventional strain gauges often lack the necessary stretchability to uniformly conform to the surface of a continuum robot. In the field of flexible electronics, carbon nanotube (CNT)–based piezoresistive fibers have emerged as a promising solution. These fibers, which offer not only high sensitivity and electrical conductivity but also substantially improved stretchability, are often used in exile wearable flexible sensors together with materials such as nylon [24,25]. CNTs, which are one-dimensional (1D) materials with a unique tubular structure, can be homogeneously mixed with polydimethylsiloxane, thermoplastic polyurethane (TPU), and other substrates in a liquid-phase environment. The resulting composite can then be processed into flexible films or fibers through dip coating, solvent evaporation, and electrospinning [26,27]. The orderly arrangement of CNTs within the substrate, along with the electron tunneling effect, endows the CNT fibers with piezoresistive properties [28,29]. The resistance of the CNT fibers exhibits a nearly linear relationship with strain within a certain range. By measuring the change in resistance, the strain of the fibers can be accurately determined, and the corresponding stress can be calculated.

Leveraging the strain-dependent resistance properties of CNT piezoresistive fibers, we have made it into an elongated, bandage-like shape, referred to as piezoresistive fiber bandage (PFB). We arranged PFB in a helical pattern on the surface of SMCR, enabling shape-sensing capabilities for existing medical continuum robots. Specifically, the bending of the robot induces strain in the PFB. By sampling the resistance changes along the robot’s body and applying data-driven methods, the shape of the continuum robot can be accurately inferred. The soft PFB is cost-effective, easy to install, and can be integrated into existing robots without modifications to their hardware design. These PFBs are lightweight, thin, and highly flexible, capable of withstanding considerable strain without compromising the robot’s stiffness or geometric dimensions. Experimental results demonstrate that this approach effectively reconstructs the shape of SMCR. In vitro and ex vivo shape-sensing experiments further indicate that this method holds promise as an ideal tool for enhancing the shape sensing capabilities of current medical continuum robots.

## Materials and Methods

### Experimental design

To evaluate the performance of the proposed system, we conducted a qualitative demonstration of typical shape sensing. In addition, the robot’s spatial shape reconstruction was measured both in vitro and ex vivo. The experimental setup consists of SMCRs incorporating CNT piezoresistive fibers, an actuation unit, in vitro models, an ex vivo model, and a PC running MATLAB/Simulink. Unless otherwise noted, all experiments were conducted at 24 °C.

In vitro experiments were performed to validate the performance of the proposed SMCR using PFB. To replicate a realistic medical scenario, phantom models were designed on the basis of computed tomography images and fabricated using 3D printing. The SMCR with PFB was inserted into these 3D-printed models, and the reconstructed shapes were compared to the known configurations. To further validate the system, shape sensing was evaluated through a comparison of simulation results and experimental data.

In the ex vivo experiments, the intestinal canal of a pig is used as the biological model. The SMCR with PFB was inserted into the real biological model, with the system’s camera providing real-time visualization of the internal environment during motion.

### Components in the sensor fabrication

The multiwalled CNTs (MCNTs) used in this experiment were purchased from Aladdin Biochemical Technology Co. Ltd. (Shanghai, China), with a diameter range of 10 to 20 nm, a length of 10 to 30 μm, and a purity of ≥95%. The *N*,*N*-dimethylacetamide (DMF) solvent was sourced from Yien Chemical Technology Co. Ltd. (Shanghai, China), with a purity of ≥99.5%. Tetrahydrofuran (THF) was purchased from MCL (Shanghai McLean Biochemical Technology Co. Ltd.). The TPU pellets, with a hardness of 60 and a particle size of 3 mm, were obtained from Guangyuan Plastics Raw Materials Co. The field-emission scanning electron microscope used for imaging was a Hitachi SU-70. The heating magnetic stirrer used in this study was the DF-101S model from Shanghai Lichen Bangxi Instrument Technology Co. Ltd. The tensile testing machine was the WH-70 model produced by Ningbo Weheng Testing Instrument Co. Ltd. The ultrasonic cleaner used was the KQ-50DB model, manufactured by Kunshan Ultrasonic Instrument Co. Ltd.

### CNT piezoresistive fiber

The fiber fabricated in this paper is 150 μm in thickness and 4 mm in width, which is consisted of TPU with 5% MCNTs. Solution evaporation is used in fabrication. The electrodes were placed uniformly on the same side of the fiber, and it is encapsulated and wrapped around the surface of the SMCR using polyurethane tape.

### Statistical analysis

Microstructural characterization of the CNT piezoresistive fiber was performed using scanning electron microscopy. Tensile testing used a universal testing machine to apply strain up to 400% at a loading rate of 1 mm/s. Fiber specimens measured 4 mm in width, 20 cm in length, and 250 μm in thickness. All tensile tests and electrical signal measurements were conducted at ambient temperature (24 °C). In vitro experiments were conducted using phantom models designed on the basis of computed tomography images and fabricated using 3D printing. The error is calculated using the Euclidean distance between the measured value and the ground truth.

## Results

### Fabrication of the sensor

**Fig. 1.**
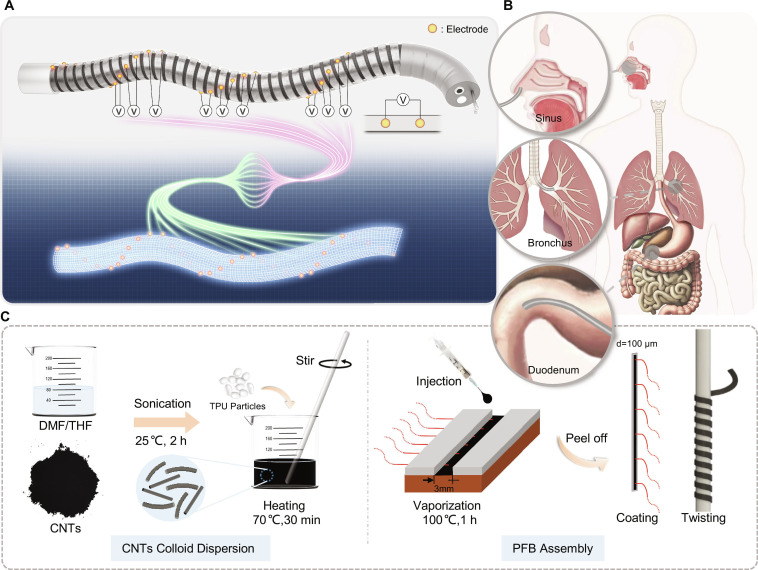
Shape sensing in slender medical continuum robot (SMCR) with piezoresistive fiber bandage (PFB). (A) Shape sensing of SMCR enabled by measuring PFB voltage in combination with a neural network. (B) Surgical scenario of SMCR includes rhinoscopy, bronchoscopy, and gastroenterostomy. (C) Schematic of CNT dispersion colloid preparation, PFB fabrication, and installation process.

To achieve uniform and dense electrode coverage on the surface of the continuum robot with PFB, we did not consider extremely fine micron-sized electrostatically spun fibers due to the difficulty of connecting multiple electrodes to individual micro- and nanofibers, which complicates wiring. To address this challenge, we made a compromise by fabricating thin, long fibrous CNT films via solution evaporation, as shown in Fig. 1C. MCNTs (0.32 g) were first dispersed in 40 ml of mixed DMF/THF solution with 1:1 wt %, using ultrasonic dispersion at 25 °C and 90% power for 6 h. TPU particles (6.4 g; 3 mm in diameter) were then added to the well-dispersed CNT solution, and the mixture was heated and stirred in a thermostatically controlled oil bath at 70 °C to melt the TPU and form a CNT colloid. A sealing film was applied to the beaker during this process to minimize evaporation of the solvents and prevent premature solidification of the TPU, as well as agglomeration of the CNTs.

In the solution evaporation step, a glass mold with a groove length of 40 cm and a depth of 1.5 mm was prepared, with adjustable width (ranging from 2 to 8 mm depending on fabrication needs) and a predesigned wiring channel. Wires were evenly spaced every 2 cm and fixed to the mold floor. Using a syringe with a diameter of 0.45 mm, the CNT colloid was injected from one end of the mold. The pressure applied through the syringe helped the CNTs form a partially ordered structure, enhancing the piezoresistive sensitivity of the PFB. Once the mold was filled, it was placed on a thermostatic heating table at 100 °C for 1 h to allow complete evaporation of the DMF and THF solvents. The resulting CNT/TPU composite film, with a thickness of 250 μm, was carefully removed from the mold.

To improve the electrode connections and enhance the sensor’s waterproof and durability properties, we used a medical-grade waterproof polyurethane adhesive film to encapsulate the entire fiber and electrode structure. To further reduce the overall resistance and fabrication complexity of the sensor, we adopted a multistage fabrication approach. The fiber sensor was produced in 3 stages, with the fabrication steps repeated 3 times to create a 3-segment sensor. This multisegment sensor was then spirally wound and fixed onto the surface of the continuum robot. After the winding process, the initial resistance of a single segment of the fiber sensor was measured to be 1 MΩ. In comparison to fiber optic sensors, the fiber resistive sensors fabricated in this study are more cost-effective, with the cost of producing and installing each sensor segment totaling less than $1.

### Characterization of the sensor

**Fig. 2.**
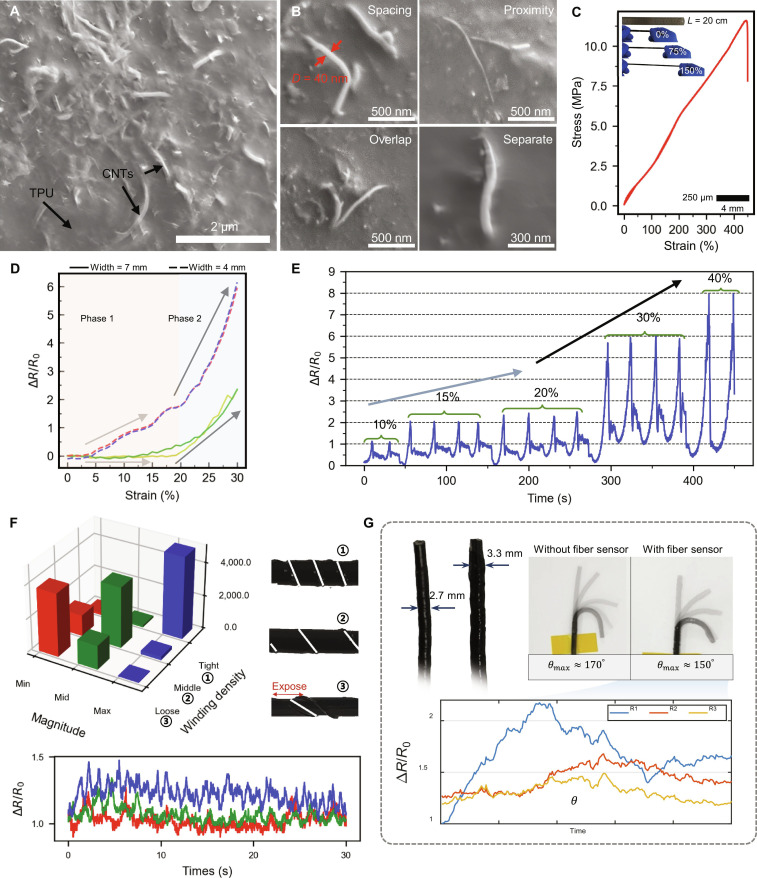
Characterization of the piezoresistive fiber bandage (PFB). (A) Scanning electron microscope (SEM) image of carbon nanotube (CNT) dispersion within the thermoplastic polyurethane (TPU) matrix. (B) SEM image showing the arrangement of CNTs within the matrix. (C) Stress–strain curve for PFB during tensile testing (inset shows manual stretching). (D) Resistance–strain relationship for PFB with different widths. (E) Variation of PFB resistance with time and strain magnitude. (F) Effect of different winding configurations on PFB sensitivity. Each dataset comprises 3 independent replicates, demonstrating the robustness and repeatability of the PFB. (G) Impact of PFB installation on the continuum robot’s performance and effect of continuum robot movement on resistance values.

The surface morphology of the sensor, as observed under a field-emission scanning electron microscope, reveals that the CNTs are uniformly distributed within the TPU substrate, as shown in Fig. 2A. The sensor was fabricated using an injection solution evaporation method, which results in 4 distinct arrangements: spacing, proximity, overlap, and separate, as illustrated in Fig. 2B. The deformation of the TPU substrate during strain application alters the contact area and arrangement of the CNTs, influencing the contact resistance and tunneling/hopping resistance, which, in turn, causes the overall resistance of the fiber to change with strain [30]. Due to the exceptional mechanical properties of CNTs, the tensile strength of the TPU matrix is enhanced. Fig. 2C demonstrates that the sensor’s stretchability reaches up to 400% without failure when tested using a tensile testing machine and, even when cut into small 8-cm sections, the sensor can be manually stretched to 150%, highlighting its excellent flexibility and confirming that it can operate under relatively low bending stresses and strains during the operation of a continuum robot.

Fig. 2C also shows the resistance–strain relationship for PFB of uniform thickness (250 μm) but different widths. The dashed line represents a 4-mm-wide sensor, while the solid line corresponds to a 7-mm-wide sensor, with different colors indicating separate test sessions. It is evident that the 4-mm-wide sensor exhibits higher sensitivity and greater consistency in resistance change between different loading cycles, making it the preferred choice for the subsequent experiments in this study. Furthermore, regardless of width, the resistance–strain curve of the fiber sensor demonstrates clear segmentation characteristics. In the strain range up to 30%, the resistance shows a nearly linear relationship with strain, while sensitivity increases notably beyond this point. Due to the relatively small strain in the spiral-wound PFB during the operation of the continuum robot, the behavior under large deformations is not explored in this paper. The experimental data indicate that a linear model cannot accurately capture the piezoresistive properties of long fiber films, such as PFBs, which informs the selection of an appropriate experimental model for future studies involving continuum robots.

Fig. 2D presents the resistance change of the PFB under continuous loading over time. The data indicate that the sensor’s resistance stabilizes under the same strain conditions, with low sensitivity below 20% strain and a marked increase in sensitivity once the strain exceeds this threshold, in agreement with the data in Fig. 2C. For more physical properties of PFB itself, please refer to Figs. S6 to S8. To identify the optimal mounting configuration of the PFB on the continuum robot, we tested 3 different winding densities, as shown in Fig. 2E. The continuum robot was controlled to trace the same trajectory within a 30-s window, and the resistance change was recorded for each winding density. Both the bar graph and the curve graph illustrate that denser winding yields higher strain sensitivity, thereby enhancing the sensor’s ability to detect bending in the continuum robot. Fig. 2F further demonstrates that using the dense winding method results in only a 0.6-mm increase in the diameter of the continuum robot and a 20° reduction in the ultimate bending angle, with negligible impact on the robot’s active bending range. The active bending section, shown by the blue line, maintains a greater range of motion, while the passive bending sections, represented by the red and yellow lines, exhibit smaller, softer movements. with all tested winding configurations performing well, these results confirm that the PFB sensor, when wound on the surface of the continuum robot, is highly effective in sensitively detecting bending motions.

### Learning the shape with the sensor data

Many studies have focused on reconstructing and representing the 3D shape of continuum robots in space. Some use Bezier curves or polynomial fittings [31], while others utilize rod models to represent the robot’s trajectory in space as a series of differential equations [32]. These modeling approaches effectively capture the robot’s shape and have demonstrated excellent results in practical applications. In this work, we adopt the widely used piecewise constant curvature (PCC) model to represent the robot’s shape in space. There are several reasons for choosing this approach in the context of our application.

First, other models often involve a large number of parameters, making it difficult to represent the robot’s specific shape using a fixed set of parameters. Second, during the process of wrapping the PFB, the electrodes were deliberately divided into 3 groups, creating narrow regions on the robot’s body that were not covered by the PFB. The stiffness variation introduced by this division naturally segments the robot into 3 parts. This allows us to directly infer the spatial arc parameters of each segment of the robot based on the resistance changes of each PFB group, effectively decoupling the robot’s spatial configuration to some extent. Finally, the PCC model has a simple mapping from the actuation space to the configuration space, which will greatly facilitate subsequent kinematic control.

FBG fibers are commonly used to measure strain at discrete points along the body of a SMCR. According to Cosserat rod theory, the shape of a continuum robot is determined by the constitutive equations of stress and strain, meaning that once the strain is measured, the shape can be reconstructed. However, FBG fibers typically require dedicated internal channels within the robot and are expensive. In the previous section, we explored the relationship between stress and strain in PFB, and their lightweight, flexible characteristics ensure minimal impact on the robot’s size and motion.

When PFBs are wrapped around the robot’s surface, the robot’s bending induces strain in the PFB, causing changes in their resistance. The degree of strain in the PFB is related to the extent of the robot’s bending at the areas where the PFB is applied. Therefore, by sampling the resistance changes across different sections of the PFB, we can indirectly infer the robot’s bending. In addition, the tightly wound spiral arrangement of the PFB, combined with downsampling through multiple electrodes, enables high sensitivity and provides sufficient information regarding the robot’s deformation in space. As a result, the proposed PFB system holds promise for a lightweight, low-cost, and easily deployable method for shape sensing of continuum robots.

The relationship between resistance changes and the robot’s shape is complex and nonlinear, further complicated by the effects of uneven assembly. This makes it a challenging problem to model. Therefore, we chose to use a data-driven approach to directly learn the robot’s shape in an end-to-end manner. To this end, we designed 3 simple fully connected neural networks, each responsible for estimating the shape of corresponding segments of the robot as mentioned earlier. The input of the neural network consists of the relative resistance changes between the electrodes, while the output is the arc parameters in a local coordinate system—specifically, the rotation plane angle ϕ and the bending angle θ.

**Fig. 3.**
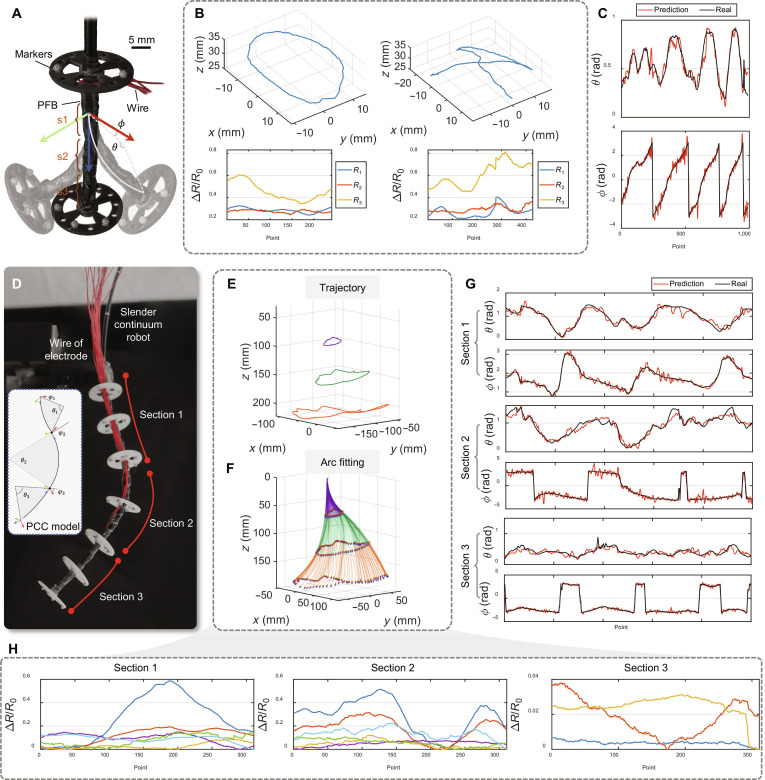
Shape sensing tests of the SMCR (slender medical continuum robot) covered with piezoresistive fiber bandage (PFB). (A) The schematic of the robot’s actuated section. (B) The robot’s end effector follows circular and cross-shaped trajectories, along with the corresponding resistance changes. (C) The performance of the neural network in perceiving the shape of a single section of SMCR. (D) The multisection constant curvature configuration diagram of the SMCR. (E) The trajectory of the robot’s 3 sections. (F) The robot shape fitted by 3 circular arcs. (G) The performance of the 3 neural networks in predicting the arc parameters. (H) The resistance changes between the corresponding electrodes of the PFB on the 3 sections of the robot.

We first conducted shape sensing tests on the last section of the SMCR, which can be actuated by pulling the tendons. As shown in Fig. 3A, a frame with small markers was installed on the robot’s body to capture real-time posture. In Fig. 3B, we controlled the robot’s end effector to move along circular and cross-shaped trajectories, while recording the relative changes in resistance across the 3 segments of the PFB. During the circular trajectory, the resistance varied in a quasi-sinusoidal pattern, directly correlating with the robot’s posture, indicating the feasibility of inferring spatial arc parameters from the resistance values.

We divided the data from a single circular arc into training, validation, and test sets in a 6:2:2 ratio, and used it to train a fully connected neural network. The trained network’s performance is shown in Fig. 3C, with an average absolute error of 3.05° for the bending angle θ and 7.49° for the rotation plane angle ϕ. For the multisection arc perception experiment shown in Fig. 3D, the 3 segments of the PFB divide the robot into 3 sections. Fifteen electrodes are arranged in the segments with 6, 6, and 3 electrodes per segment, respectively. In Fig. 3E, we manually operated the robot along an irregular closed trajectory using thin lines that did not obstruct the markers. We then fitted the robot’s shape using 3 circular arcs of equal length, as shown in Fig. 3F. Using the same method, we collected approximately 10,000 data points and trained 3 separate neural networks. The performance of these networks is shown in Fig. 3G, with average absolute errors of 5.05° for the bending angle θ and 13.23° for the rotation plane angle ϕ. The resolution of shape perception is 0.5° for both bending angle θ and rotation plane angle ϕ. The detection range spans 0° to 90° for θ and 0° to 360° for ϕ, with minimum detectable changes of 0.5° and 0°, respectively.

### In vitro and ex vivo experiments

In vitro experiments were conducted to assess the effectiveness of shape sensing in the SMCR system using PFB. The robot was mounted on a custom-designed test platform that enabled precise teleoperated control of the distal end, resulting in passive deformation of the proximal segment. In vitro experiment involved inserting the SMCR into 3 transparent phantoms that closely replicate human tissue. The PFB was integrated with a voltage measurement system to capture the resistance changes corresponding to the deformation of the robot segments. These resistance variations provided real-time feedback on the morphological changes of the entire SMCR.

**Fig. 4.**
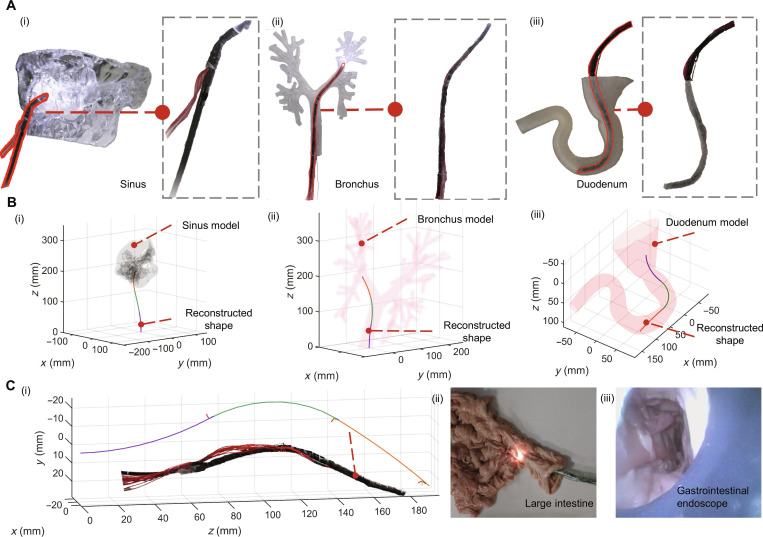
Shape sensing in in vitro and ex vivo experiments. (A) Schematics of the in vitro experiments using (i) sinus, (ii) bronchial, and (iii) duodenal phantoms. (B) Shape estimation results in the (i) sinus, (ii) bronchial, and (iii) duodenal phantoms. The SMCR is positioned within the narrow cavities of the tissues, with the estimation results consistent with the actual configurations. (C) Ex vivo experiment in a pig large intestine: (i) estimated SMCR shape and (ii, iii) external and internal views of the experimental setup.

A series of in vitro experiments were conducted in 3 distinct scenarios: (a) sinus model, with bending restricted to the distal segment; (b) bronchus model, with bending of both the distal and middle segments at varying angles; and (c) duodenum model, where 3 segments bent into an “S” shape. Corresponding real shapes for each case are shown in Fig. 4A. As illustrated in Fig. 4B, shape reconstruction was performed in MATLAB. The resistance data collected from the PFB were input into the well-trained neural network, achieving the shape sensing of SMCR.

To demonstrate the potential clinical application of SMCR with PFB, we carried out ex vivo studies. Given the anatomical similarities of porcine tissue to human tissue, particularly in terms of soft tissue elasticity and mechanical properties, the intestinal canal of a pig was chosen as the model. The SMCR was inserted into the porcine model, simulating typical medical procedures such as sinus surgery, bronchoscopy, and gastrointestinal endoscopy. PFB sensor feedback was utilized to adjust the robot’s position throughout the procedure.

As shown in Fig. 4C, the SMCR with PFB successfully performed gastrointestinal endoscopy in the porcine intestine, the proposed sensing system providing visualization of the canal. The performance of the device was shown to be adaptable to ex vivo conditions, demonstrating the feasibility of using the proposed system in medical applications.

## Discussion

In this paper, we developed a PFB that achieves high sensitivity in the small strain range, which can be spirally wound onto the surface of an SMCR, enabling its shape-sensing ability. The resistance changes between uniformly distributed electrodes are converted into shape configurations by several well-trained neural networks. The fabrication process of PFB is simple and cost-effective and offers excellent stretchability, with a thin profile and adjustable length during fabrication to meet specific needs, which will lead to a negligible impact on the stiffness and motion performance of the continuum robot.

Comprehensive material property characterization and shape perception experiments have demonstrated that the stretchable, high-sensitivity PFB can endow existing SMCR with the ability to perceive their shape. In vitro experiments, including endoscopic simulations of the nasal sinus cavity, pulmonary bronchi, and duodenum, as well as exploratory tests inside the ex vivo colon of a pig, reveal the broad medical application potential of PFB for robot shape perception. This capability will enhance a surgeon’s control over the continuum robot that cannot be directly visualized inside the patient’s body, ultimately improving surgical outcomes.

It is worth noting that the current prototype, due to the presence of multiple electrodes, results in excessive wiring at the robot’s base side, which limits its movement to some extent. In addition, the PCC model remains simplistic and can lead to substantial shape reconstruction errors in certain regions of the robot’s body. Although in vitro experiments validate the feasibility of the proposed approach, several practical issues related to medical deployment remain open, including long-term drift, cyclic durability, sterilization compatibility, and wiring scalability. These aspects are beyond the scope of the current study and will require further systematic investigation and engineering development in future work. In future work, we plan to further streamline the electrical wiring of the PFB and adopt more precise models to better describe the robot’s shape in space. In addition, we will continue to explore further functionalities of the PFB sensor, such as utilizing the resistance–strain relationship for force sensing and force-feedback control of the continuum robot.

## Data Availability

All data needed to evaluate the conclusions in the paper are present in the paper and/or the Supplementary Materials. Additional data related to this paper may be requested from the authors.

## Supplementary Materials

Supplementary 1 Discussions S1 to S4 Figs. S1 to S8 Movie S1
