# Supplementary material for: Enhancing Shape Sensing of Slender Medical Continuum Robot Using Carbon Nanotube Piezoresistive Fiber Bandage
Source: Cyborg Bionic Syst. 2026 Jun 24;7:0622. doi: 10.34133/cbsystems.0622 (PMC13291483; doi:10.34133/cbsystems.0622)
Supplement: Supplementary 1 — Discussions S1 to S4 Figs. S1 to S8 Movie S1 [file cbsystems.0622.f1.zip › Supplementary Material.docx]

**Supplementary Information for**

**Enhancing Shape Sensing of Slender Medical Continuum Robot Using CNTs Piezoresistive Fiber Bandage**

Pingyu Xiang^1^†, Xiangyu Mi^1^†, Hongye Zhang^1^†, Fei Wang^1^†, Xiong Yang^2^, Yue Wang^1^, Rong Xiong^1^, Song Liu^3^, Haojian Lu^1,4,5^*

^1^Department of Control Science and Engineering, Zhejiang University; Hangzhou 310027, China.

^2^Department of Electronic and Computer Engineering, The Hong Kong University of Science and Technology.

^3^School of Information Science and Technology, ShanghaiTech University, Shanghai 201210, China.

^4^Stomatology Hospital, School of Stomatology, Zhejiang University School of Medicine, Zhejiang Provincial Clinical Research Center for Oral Diseases, Zhejiang Key Laboratory of Oral Biomedical, Hangzhou 310000, China.

^5^Engineering Research Center of Oral Biomaterials and Devices of Zhejiang Province, Hangzhou 310000, China.

†These authors contributed equally to this work

*Corresponding author. Email: luhaojian@zju.edu.cn

**The PDF file includes:**

Supplementary Discussion 1 to 4

Supplementary Fig. 1 to 8

Supplementary Video 1

**Table of Contents**

[Supplementary Discussions 3](#_Toc228566529)

[Supplementary Discussion 1. Piecewise Constant Curvature Model 3](#_Toc228566530)

[Supplementary Discussion 2. Structure of Slender Medical Continuum Robot 3](#_Toc228566531)

[Supplementary Discussion 3. *In-vitro* and *ex vivo* experiment 4](#_Toc228566532)

[Supplementary Discussion 4. Data acquisition 4](#_Toc228566533)

[Supplementary Figures 6](#_Toc228566534)

[Supplementary Fig. 1 | Schematic of Slender Medical Continuum Robot 6](#_Toc228566535)

[Supplementary Fig. 2 | *In vitro* and *ex vivo* phantom experiment setup 6](#_Toc228566536)

[Supplementary Fig. 3 | PFB Data Acquisition Circuit 7](#_Toc228566537)

[Supplementary Fig. 4 | Schematic diagram of the PFB neural network. 7](#_Toc228566538)

[Supplementary Fig. 5 | Schematic diagram of PCC arc parameters. 8](#_Toc228566539)

[Supplementary Fig. 6 | Effect of temperature on resistance. 8](#_Toc228566540)

[Supplementary Fig. 7 | Caption Effect of bending angle on resistance. 9](#_Toc228566541)

[Supplementary Fig. 8 | Comparison of stress-strain curves for TPU and TPU/CNTs during tensile testing. 9](#_Toc228566542)

[Supplementary Video 9](#_Toc228566543)

# Supplementary Discussions

## Supplementary Discussion 1. Piecewise Constant Curvature Model

The Piecewise Constant Curvature (PCC) model is a method used in robotics and mechanical engineering to represent the shape and motion of flexible or continuum robots. In this model, the robot or structure is divided into multiple segments, and each segment is assumed to have a constant curvature. The curvature remains unchanged within each segment but can differ between segments, allowing the robot to approximate complex shapes and movements.

In the coordinate system defined by the figure, the coordinates can be expressed as:

$$x_{rot}=\left[ \begin{matrix} v_{rot} \\ w_{rot} \end{matrix} \right]={[\begin{matrix} \begin{matrix} 0 & 0 & 1 \end{matrix} & \begin{matrix} 0 & 0 & 0 \end{matrix} \end{matrix}]}^{T}$$

$$x_{inp}=\left[ \begin{matrix} v_{rot} \\ w_{rot} \end{matrix} \right]={[\begin{matrix} \begin{matrix} 0 & Ϗ \end{matrix} & \begin{matrix} 0 & 0 \end{matrix} & \begin{matrix} 0 & l \end{matrix} \end{matrix}]}^{T}$$

Where$\boldsymbol{v}$ and ***w*** represent linear differential motion and angular differential motion, respectively.$Ϗ=l/\theta$denotes the curvature of the segment of the arc, $x_{inp}$ indicating rotation about the z-axis, while $x_{\mathrm{inp}}$represents the transformation along the central axis. Writing this coordinate as a matrix yields:

$$x_{rot}^{\wedge}=\left[ \begin{matrix} \omega_{rot}^{\wedge} & v_{rot} \\ 0 & 0 \end{matrix} \right]=\left[ \begin{matrix} \begin{matrix} 0 & -1 \\ 1 & 0 \end{matrix} & \begin{matrix} 0 & 0 \\ 0 & 0 \end{matrix} \\ \begin{matrix} 0 & 0 \\ 0 & 0 \end{matrix} & \begin{matrix} 0 & 0 \\ 0 & 0 \end{matrix} \end{matrix} \right]$$

$$x_{inp}^{\wedge}=\left[ \begin{matrix} \omega_{inp}^{\wedge} & v_{inp} \\ 0 & 0 \end{matrix} \right]=\left[ \begin{matrix} \begin{matrix} 0 & 0 \\ 0 & 0 \end{matrix} & \begin{matrix} Ϗ & 0 \\ 0 & 0 \end{matrix} \\ \begin{matrix} -Ϗ & 0 \\ 0 & 0 \end{matrix} & \begin{matrix} 0 & 1 \\ 0 & 0 \end{matrix} \end{matrix} \right]$$

Where the operator $\wedge$ represents the mapping from $\boldsymbol{R}^{3}$ to $so(3)$ (the Lie algebra), and the operator ∨ is its inverse.

Using the matrix exponential, it can be shown that:

$$T=e^{(x_{rot}^{\wedge}\phi)}e^{(x_{inp}^{\wedge}l)}$$

$T$ is the coordinate transformation matrix for the arc. Similarly, the expression can also be replaced by:

$$T=e^{(\hat{\boldsymbol{x}})}$$

Where $\boldsymbol{x=}{[\begin{matrix} \begin{matrix} -Ϗlsin\phi& Ϗlcos\phi\end{matrix} & \begin{matrix} 0 & 0 \end{matrix} & \begin{matrix} 0 & l \end{matrix} \end{matrix}]}^{T}$

In this paper, the SMCR is regarded as three segments of constant curvature arcs, so its end-effector pose can be written as:

$$T=e^{(\boldsymbol{x}_{1}^{\wedge})}e^{(\boldsymbol{x}_{2}^{\wedge})}e^{(\boldsymbol{x}_{\boldsymbol{3}}^{\boldsymbol{\wedge}})}$$

Where $\boldsymbol{x}_{\boldsymbol{i}}\boldsymbol{=}{[\begin{matrix} \begin{matrix} -Ϗ_{i}l_{i}sin\phi_{i} & Ϗ_{i}l_{i}cos\phi_{i} & 0 \end{matrix} & \begin{matrix} 0 & 0 & l_{i} \end{matrix} \end{matrix}]}^{T}$

## Supplementary Discussion 2. Structure of Slender Medical Continuum Robot

The structure of SMCR we conducted the experiments are 3.6mm in diameter and 600mm in length. There is a 30mm continuum part as distal parts could be actuated by a linear motor for motion, consisting of vertically arranged joints. As for the proximal part, it is passive, but it will be affected by the environment and the movement of the distal end, thus it will also produce a certain shape change. Near-end steering is controlled by manipulating teleoperation equipment.

## Supplementary Discussion 3. *In-vitro* and *ex vivo* experiment

The experimental platform consists of slender medical continuum robots (SMCR)) with Piezoresistive Fiber Bandage (PFB), an actuation unit, in vitro models, a vivo model, and a PC with MATLAB/Simulink.

**Slender medical continuum robots** (**SMCR)**: The structure of slender medical continuum robots we conducted the experiments are 3.6mm in diameter and 600mm in length.

**Piezoresistive Fiber Bandage (PFB)** : The fiber fabricated in this paper is 150 μm in thickness and 4 mm in width, which is consisted of TPU with 5% MCNTs. Solution evaporation is used in fabrication. The electrodes were placed uniformly by on the same side of the fiber and it is encapsulated and wrapped around the surface of the slender medical continuum robot using PU tape.side of the fiber and it is encapsulated and wrapped around the surface of the slender medical continuum robot using PU tape.

**Actuation unit**: It includes four linear motors (LA50-021D, Inspire-Robots, Beijing, China) to connect four tendons of the catheter in the SMCR for motion.

**Vitro and vivo model**: The model of the maxillary sinus, bronchus, and intestinal canal were used for vitro experiment. According to the CT data from a normal adult, synthetic phantoms were constructed to simulate the mechanical properties of biological tissues.

The model of the intestinal canal was used for the vivo experiment.

**PC**: The control system consists of a personal computer. The proposed shape sensing algorithm and control software integrated into Microsoft Visual Studio serves as the central station for controlling the surgical robot.

The shape of SMCR and the phantom models were simulated in Matlab to compare with the real experiment.

## Supplementary Discussion 4. Data acquisition

To obtain the ground truth of the shape of the SMCR, retroreflective markers were uniformly attached to the robot. A motion capture system was used to collect key points from the robot's 3D spatial configuration, which were considered the true shape of the robot. These key points were then used to evaluate the error in shape estimation and to serve as a training dataset for learning algorithms.

To support the operation of the Motive software, we set up a motion capture space measuring 2 m in height, 3 m in length, and 1.7 m in width. Five OptiTrack PRIME17W high-frame-rate cameras were used to capture the optical signals from the retroreflective markers attached to the slender medical continuum robot. Power over Ethernet (PoE) cables were used to supply power to the cameras, while they were connected to the host computer via a NETGEAR switch.

# Supplementary Figures


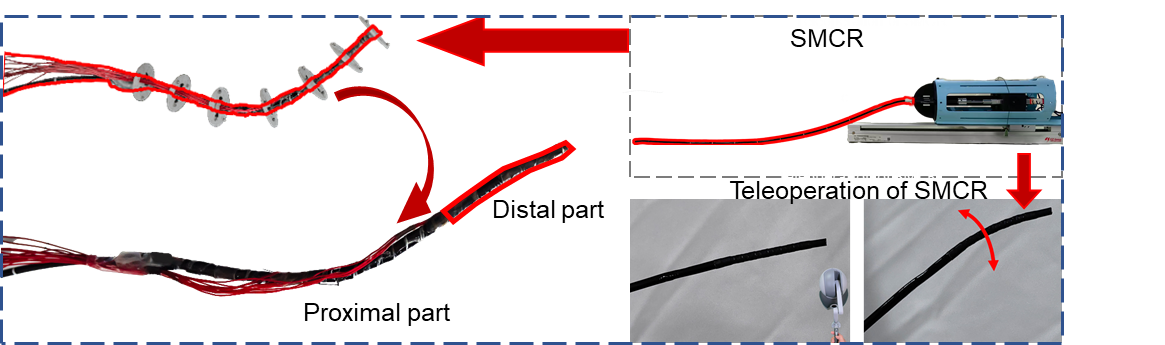


## Supplementary Fig. 1 | Schematic of Slender Medical Continuum Robot

Structure detail of slender medical continuum robot including teleoperation, distal part and proximal part.


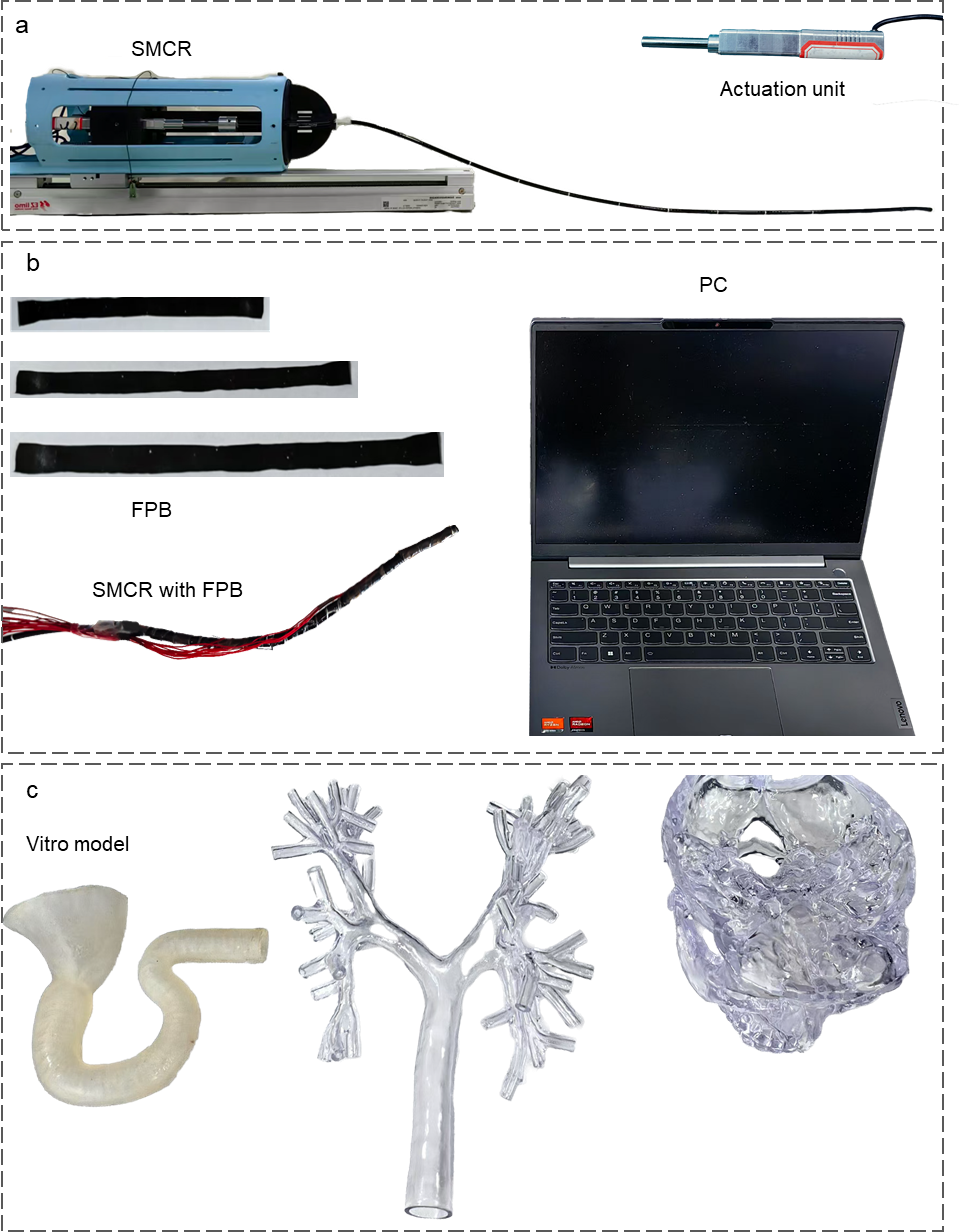


## Supplementary Fig. 2 | *In vitro* and *ex vivo* phantom experiment setup

**a,** Slender Medical Continuum Robot with actuation unit. **b,** Actual operating system with FPB and PC. **c,** *In vitro* model includes the maxillary sinus, bronchus, and duodenum.


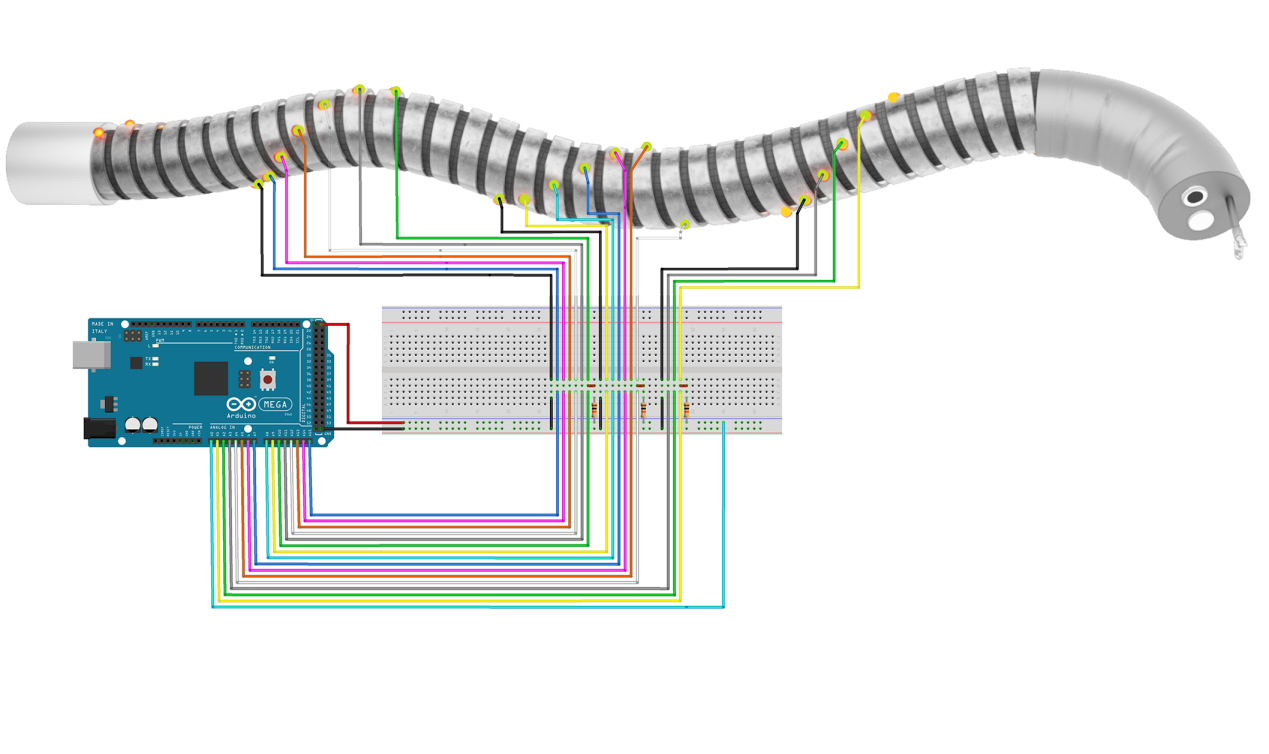


## Supplementary Fig. 3 | PFB Data Acquisition Circuit

The data acquisition circuit composed of PFB and Arduino collects the voltage on the PFB electrodes to determine the PFB resistance data.


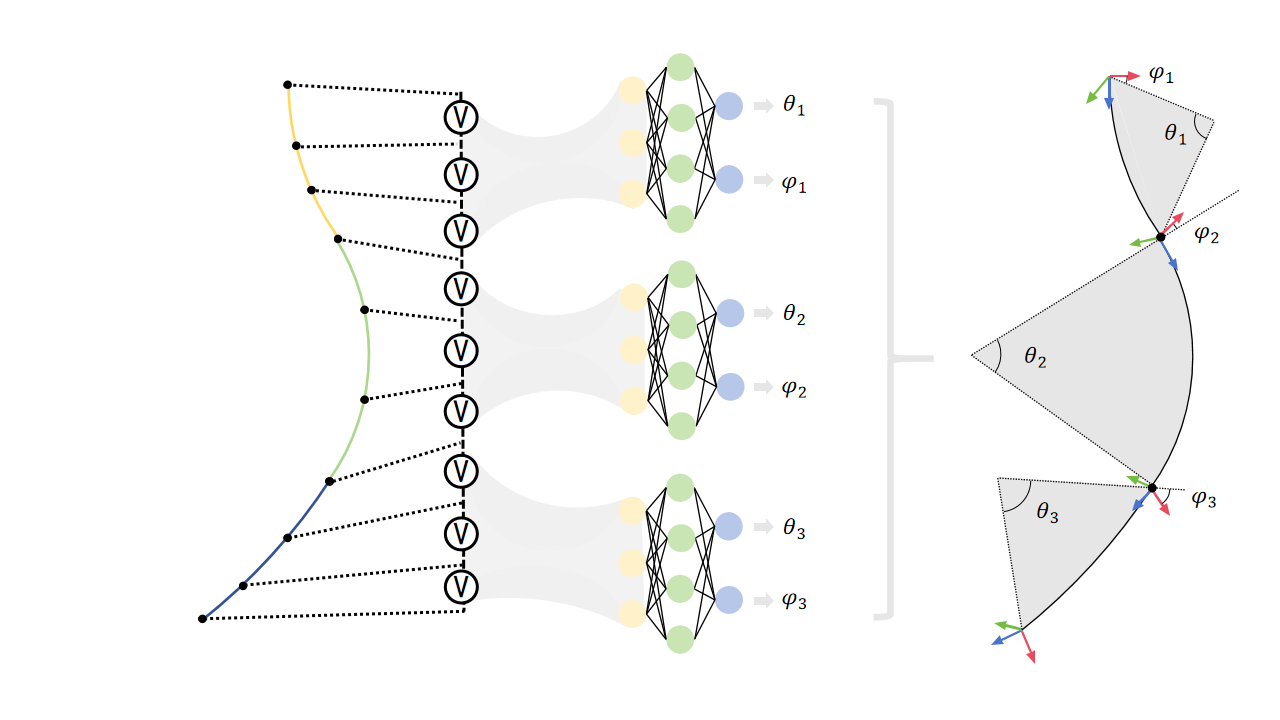


## Supplementary Fig. 4 | Schematic diagram of the PFB neural network.


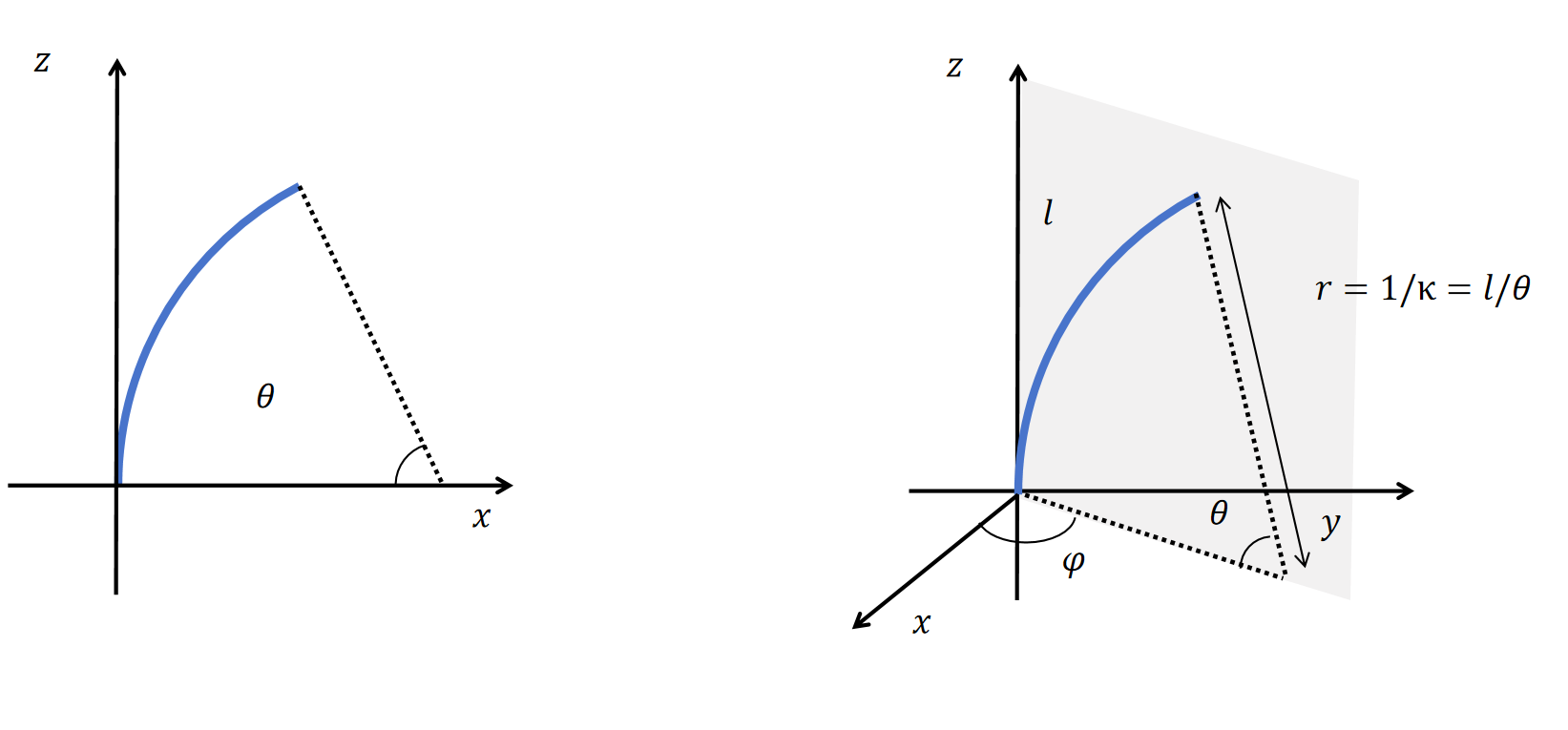


## Supplementary Fig. 5 | Schematic diagram of PCC arc parameters.


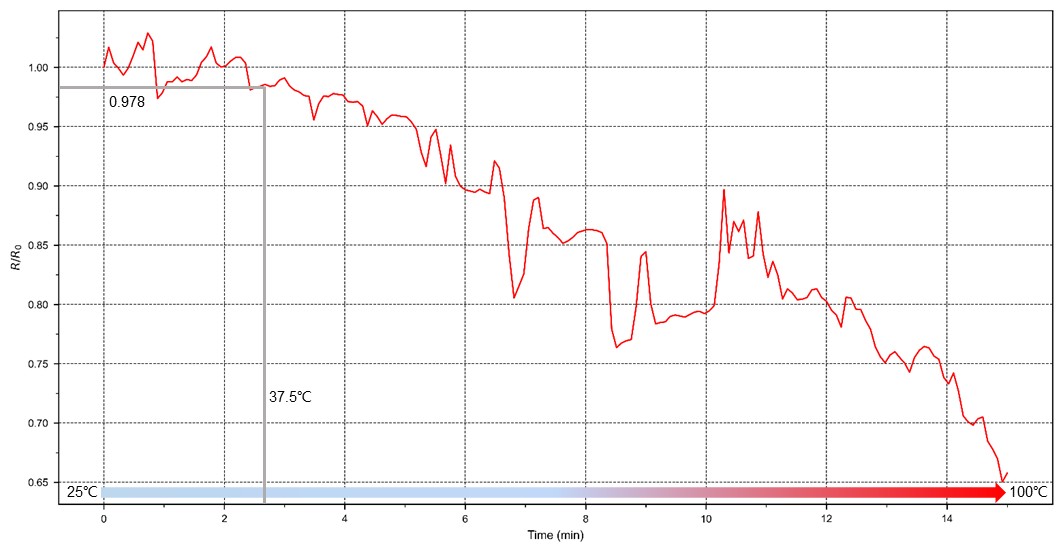


## Supplementary Fig. 6 | Effect of temperature on resistance.

Within 15 minutes, the PFB was uniformly heated from room temperature (25°C) to 100°C using a thermostatic heater. Overall, the resistance of the PFB exhibited a downward trend as the temperature increased. However, as indicated by the gray line in the figure, when the temperature reached 37.5°C—the maximum normal body temperature—the resistance decreased to only 97.8% of its original value. In contrast, under bronchoscope operating conditions, the average resistance variation exceeds 50%, making this decrease negligible. Therefore, the PFB developed in this study is suitable for use under in vivo conditions required for SMCR.


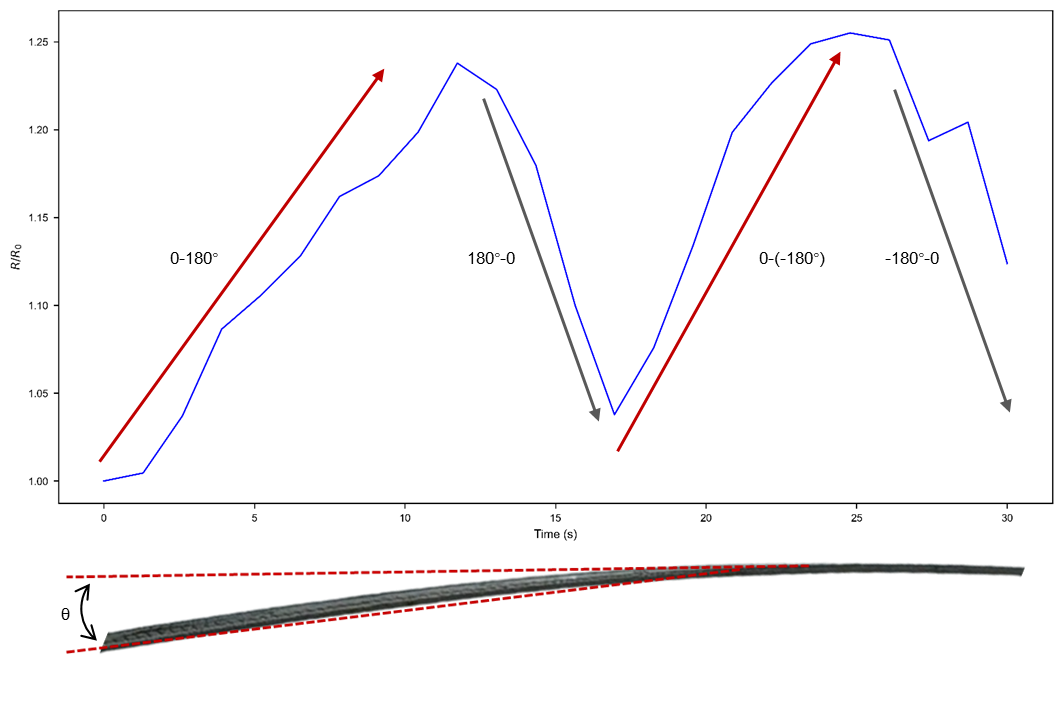


## Supplementary Fig. 7 | Caption Effect of bending angle on resistance.

By adhering the PFB to an insulated metal ruler, the PFB effectively senses changes in the bending angle (θ) of the ruler.


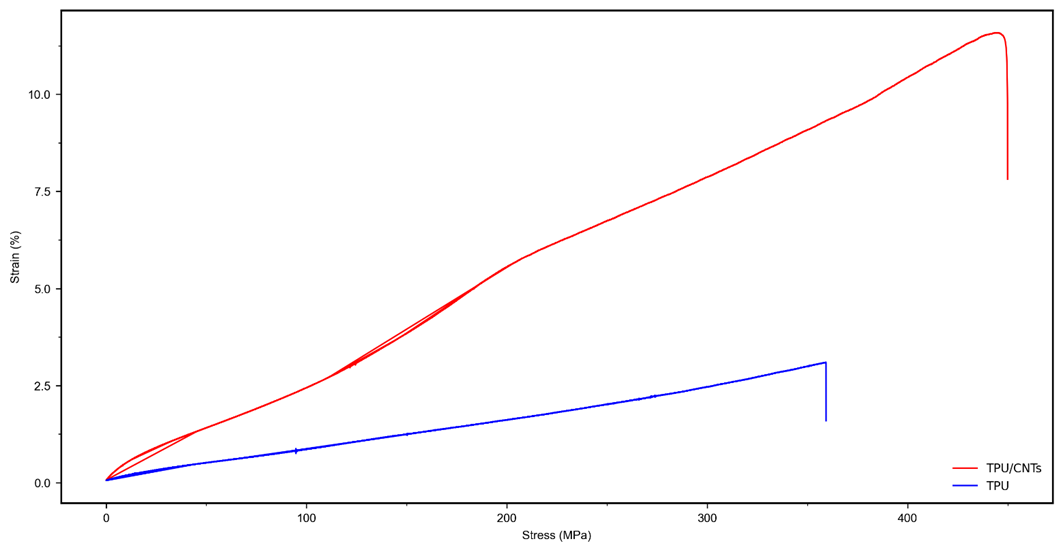


## Supplementary Fig. 8 | Comparison of stress-strain curves for TPU and TPU/CNTs during tensile testing.

The addition of CNTs enhances the physical properties of the TPU, resulting in reduced viscosity of the TPU matrix and improved mechanical performance.

# Supplementary Video

This video demonstrates the basic performance of the bandage and its shape-sensing capability after integration with the continuum robot.
